# A Possible Indicator of Oxidative Damage in Smokers: (13*Z*)-Lycopene?

**DOI:** 10.3390/antiox6030069

**Published:** 2017-09-13

**Authors:** Daniel L. Graham, Mario Lorenz, Andrew J. Young, Gordon M. Lowe

**Affiliations:** 1 Faculty of Science, Liverpool John Moores University, Byrom Street, Liverpool L3 3AF, UK; d.l.graham@ljmu.ac.uk; 2 Charité—Universitätsmedizin Berlin, corporate member of Freie Universität Berlin, Humboldt-Universität zu Berlin, and Berlin Institute of Health, Department of Cardiology and Angiology, CCM, Charitéplatz 1, 10117 Berlin, Germany; mario.lorenz@charite.de; 3 School of Natural Sciences and Psychology, Liverpool John Moores University, Byrom Street, Liverpool L3 3AF, UK; a.j.young@ljmu.ac.uk; 4 School of Pharmacy and Biomolecular Sciences, Liverpool John Moores University, Byrom Street, Liverpool L3 3AF, UK

**Keywords:** carotenoid, isomerization, lycopene, smoking, tomato

## Abstract

In vitro, the gaseous phase of cigarette smoke is known to induce both isomerization and degradation of dietary carotenoids, such as β-carotene and lycopene. However, the effects of cigarette smoke on the composition of circulating lycopene in vivo are not well understood. In this study, we examined the lycopene profiles of plasma from non-smokers and smokers. No oxidative intermediates of lycopene that have been observed previously in vitro were detected in the plasma, but evidence of isomerization of the carotenoid was seen. Four geometric forms of lycopene were detected in the plasma of both smokers and non-smokers, namely the (5*Z*), (9*Z*), (13*Z*) and (all-*E*) forms. The relative amounts of these isomers differed between the two cohorts and there was a significant difference (*p* < 0.05) between smokers and non-smokers for the ratio of total-Z:all-*E* lycopene, and in the relative amounts of (13*Z*) and (all-*E*)-lycopene. The ratio of (all-*E*):(13*Z*)-lycopene was 0.84:1.00 in smokers compared to 1.04:1.00 in non-smokers. In smokers, the (13*Z*)-isomer was generated in preference to the more thermodynamically stable (5*Z*) and (9*Z*)-isomers. This mirrors the scenario seen in vitro, in which the formation of (13*Z*)-lycopene was the main isomer that accompanied the depletion of (all-*E*) lycopene, when exposed to cigarette smoke. The results suggest that the relative amount of (13*Z*)-lycopene could be used as an indicator of oxidative damage to lycopene in vivo.

## 1. Introduction

A diet rich in fruit and vegetables is generally thought to be associated with a lower risk of developing certain cancers and other chronic diseases [1,2,3]. In recent years, this view has been increasingly challenged, due to conflicting information arising from studies on alcohol consumption and cigarette smoking, leading to the assumption that a dramatic increase in the intake of fruit and vegetables does not significantly decrease the relative risk of developing chronic disease [4,5]. One observation that has been made is that cigarette smokers generally have a lowered plasma concentration of both water- and lipid-soluble antioxidants, including carotenoids, derived from their diet, compared to non-smokers [5,6]. This could be due to decreased consumption of fruit and vegetables, or the impact of smoke on absorption or oxidation of carotenoids. However, some reports do highlight an inadequate diet in smokers, e.g., [7]. Levels of both carotenes and xanthophylls in the plasma are recognised as good biomarkers for the consumption and dietary absorption of plant pigments [8].

The gaseous phase of cigarette smoke contains many constituents, including nitric oxide and peroxynitrite [9]. It has been suggested that the gaseous oxidants present in cigarette smoke may cross the alveolar cells of the lung and enter the bloodstream [10]. Evidence for this has been observed through changes in the membranes of erythrocytes or modification to low density lipoproteins (LDL) [11,12]. Lycopene is primarily transported in the bloodstream bound to LDL particles [13], and has only rarely been detected in high density lipoproteins (HDL) particles, at very low levels [14].

**Figure 1:**
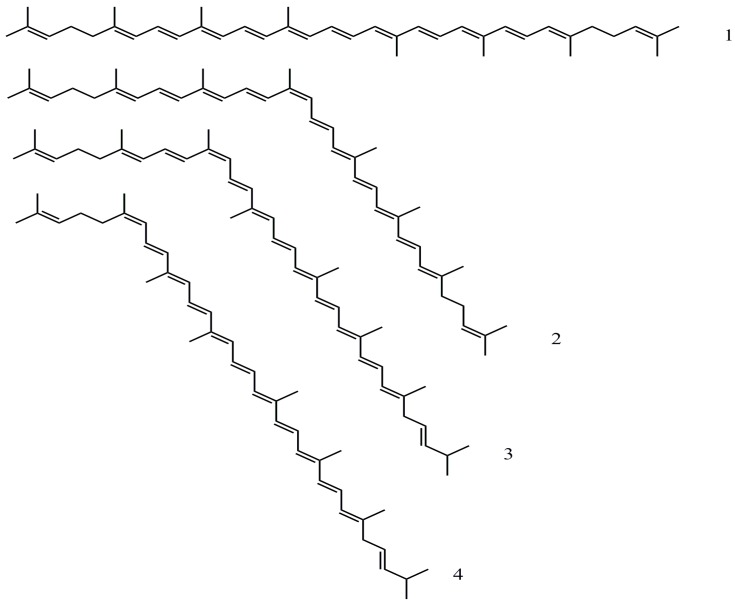
Geometrical forms of lycopene detected in human plasma exposed to cigarette smoke: (**1**) (all-*E*)-lycopene; (**2**) (13*Z*)-lycopene; (**3**) (9*Z*)-lycopene; (**4**) (5*Z*) lycopene.

Along with several other circulating antioxidants, carotenoid concentrations in the human plasma have generally been reported as being lower in smokers [6,15,16]. While levels of β-carotene (β,β-carotene) are consistently lower in smokers (by ca. 25%) the pattern is less clear for lycopene (Ψ,Ψ-carotene) [17]. In a recent in vitro study [18], cigarette smoke was gently bubbled through human plasma and isolated LDL. In both scenarios, depletion of both lycopene and β-carotene was seen, along with changes to the geometric conformation of the lycopene molecule, i.e., isomerisation. In tomato products and other foods, such as watermelon, lycopene exists mainly as (all-*E*)-lycopene, but in plasma it exists as a mixture of (*Z*)-isomers, including the (5*Z*)-, (9*Z*)- and (13*Z*)- forms (Figure 1) [19]. When plasma isolated from non-smokers was challenged with cigarette smoke, isomerisation of (all-*E*)-lycopene (the dominant form of this carotenoid found in plasma or LDL) took place. As a result of this, the ratio of (all-*E*):(*Z*) isomers of lycopene was altered. A wide range of reaction products of β-carotene [20] and lycopene [19] are formed as a result of the interaction with these components of cigarette smoke. In vitro, the (13*Z*)- and (9*Z*)-isomers of lycopene are major intermediates in the oxidative degradation of lycopene by cigarette smoke, and also by Sin-1 (S-morpholinosydonimine; a component of cigarette smoke and a generator of nitric oxide and superoxide). A number of epoxides of lycopene (e.g., lycopene-1,2-epoxide) are also formed due to the interaction with the conjugated double bond chain. Graham et al. [1] further demonstrated that in isolated human plasma, levels of (all-*E*)-lycopene are depleted to a greater extent than levels of (5*Z*)-lycopene or β-carotene, while in isolated LDL, both (all-*E*)- and (5*Z*)-lycopene were shown to be more susceptible than β-carotene. Such results might support the role of the sacrificial regenerative properties of lycopene in protecting other antioxidants.

Components from the gaseous phase of cigarette smoke are known to cross the alveolar wall and enter the bloodstream [10,12]. However, despite evidence that cigarette smoke has a deleterious effect on carotenoids in vitro (including isomerisation and degradation), the effects of cigarette smoke on the isomeric composition of circulating lycopene in vivo are not well understood. 

## 2. Materials and Methods

### 2.1. Study Design

This study was approved by the Research Ethics Committee of Liverpool John Moores University (09/SPS/037). Fifty-two healthy volunteers, aged 21–65, were recruited for the survey. Exclusion criteria included the following: use of any prescribed medication, a cardiovascular risk (e.g., high plasma cholesterol, diabetes or hypertension), a bleeding disorder or pregnancy.

All participants answered a simple questionnaire requesting details about their age, number of cigarettes smoked per day, height and weight, as well as some general questions concerning their dietary consumption of processed tomato products.

### 2.2. Carotenoid Analysis

Blood was taken by venipuncture (after overnight fasting) and mixed with 3.8% (w/v) tri-sodium citrate at a ratio of one part anticoagulant to nine parts blood. Plasma was obtained by centrifugation at 500× *g* for 15 min, removed using a plastic pipette, and where necessary, stored at −20 °C until required.

Carotenoids were extracted from both plasma and LDL samples as follows: 1 mL samples were added to a glass vial, to which 1 mL of ethanol was added. The sample was vortexed immediately for 2 s, and 1.5 mL of diethyl ether was added. The sample was vortexed again (2 s), after which 1.5 mL of hexane was added. The sample was vortexed (2 s) and allowed to stand until the sample displayed a clear partition. The upper layer was removed with a glass Pasteur pipette and dried in an amber vial under oxygen-free nitrogen (OFN). When the sample was completely dry, it was re-suspended in 100 µL of tetrahydrofuran and 400 µL of methanol. This was transferred to an amber vial for analysis by high-performance liquid chromatography (HPLC). Samples were usually analyzed immediately, but if this was not possible they were stored dry under OFN at −20 °C for a maximum of 24 h.

### 2.3. High-Performance Liquid Chromatography 

HPLC was performed using an Agilent 1100 series system, comprising a binary pump and diode array detector. All samples were analyzed twice using C18 and C30 reversed-phase columns. This permitted optimal separation of components in order to detect carotenoids and the geometric isomers of lycopene. For the initial analysis, 50 µL of sample (in tetrahydrofuran:methanol, 1:4, v/v) was injected onto a C18 reversed-phase column (5 µm particles, 4.6 × 250 mm Gemini, Phenomenex, Macclesfield, UK) at room temperature. The mobile phase (66/22/10 acetonitrile/tetrahydrofuran/methanol plus 0.005% w/v ammonium acetate) was delivered at a rate of 0.8 mL/min. For the analysis of the geometrical isomers of lycopene, a C30 column (5 µm particles, 4.6 × 250 mm YMC, VWR, Lutterworth, UK) was operated at a constant temperature of 40 °C. The mobile phase was methyl-tert-butyl-ether/methanol/ethyl acetate (50/40/10 v/v/v) delivered isocratically at 0.45 mL/min. For both systems, detection was done by diode array detection in the range of 300–600 nm and integration of each peak was performed using Chemstation software (Agilent, v10A, Stockport, UK). Lycopene isomers were identified by their on-line spectra and retention times in comparison to authentic standards (kindly provided by Dr. V. Böhm, University of Jena).

### 2.4. Lipid Profiles

Plasma cholesterol, LDL cholesterol, apo B-100, apo A1, small dense LDL, and triglyceride assays were all perfomed on the RX Daytona clinical analyser (Randox, Laboratories Ltd., Antrim, UK). All assays were performed with authentic standards and controls. 

### 2.5. Oxidised LDL

The concentration of oxidised LDL in plasma was determined using an enzyme linked enzyme immunosorbent assay, using a commercially available kit (Mercodia AB, Uppsala, Sweden).

### 2.6 Statistics

Experimental results are expressed as mean ± standard deviation (SD). The data was analyzed using the student’s *t*-test and a one-way analysis of variance. Statistical significance was defined as *p* < 0.05. Normal distribution of the data was assessed using a Shapiro-Wilk test, and the equality of variance was determined using the Lavene’s test. All statistics were performed using SPSS v 23 (IBM, Portsmouth, UK).

## 3. Results

The main clinical and biochemical characteristics of the smoking and non-smoking groups are presented in Table 1. The lipid profiles of the smokers suggest a more atherogenic profile, with elevated LDL cholesterol and lower HDL cholesterol concentrations compared to the non-smoking group (*p* < 0.05). The plasma from all the participants was also screened to determine circulating β-carotene and lycopene concentrations (Table 2). Plasma levels for these two carotenoids were in the range of 0.4–0.7 µmol/L. There were no significant differences in the total amounts of either carotenoid between the smoking and non-smoking cohorts.

**Figure 2:**
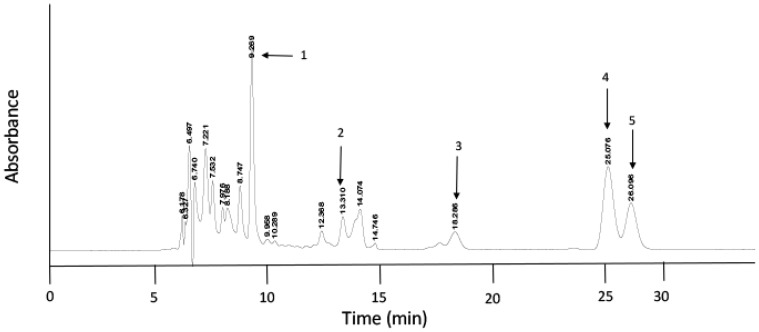
HPLC elution profile recorded at 460 nm showing the separation of the β-carotene and the geometric isomers of lycopene from an extract of plasma taken from a non-smoker:β-carotene (1); (13*Z*)-lycopene (2); (9*Z*)-lycopene (3); (all-*E*)-lycopene (4); and (5*Z*)-lycopene (5). The details for the extraction and separation are described in the methods section.

**Figure 3:**
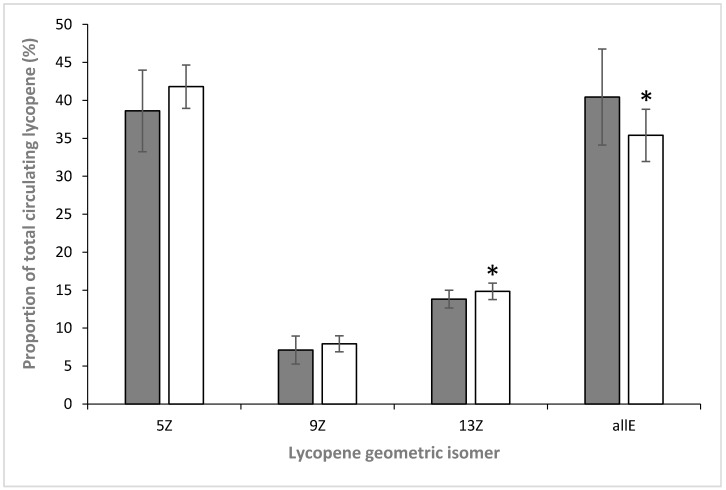
Changes in the relative amounts of individual geometrical isomers of lycopene, expressed as a percentage of total circulating lycopene in smokers (clear) and non-smokers (shaded). *n* = 52 ± SD * indicates significant differences between smokers and non-smokers (*p* < 0.05).

HPLC analysis, using a C30 reversed-phase system, was used to determine the profile of lycopene geometric isomers in plasma (Figure 2). In order to normalise the dataset, the relative proportion of each isomer was determined with respect to the total lycopene concentration in each individual (Table 2, Figure 3). This showed that (all-*E*)-lycopene was the most abundant geometrical form of this carotenoid in the plasma of all participants (smokers and non-smokers). The (all-*E*) form was accompanied by the presence of several geometric isomers of the carotenoid, namely (5*Z*), (9*Z*) and (13*Z*) lycopene (Figure 1), which were identified by a combination of their chromatographic and spectral characteristics (Table 3). Of these, the (5*Z*) form was the most abundant, with circulating levels comparable to those of the (all-*E*)-isomer. Together with the (all-*E*) form, this accounted for approximately 80% of the total lycopene. The (9*Z*) and (13*Z*) forms accounted for the remainder of the circulating lycopene, with the (13*Z*) form being approximately twice as abundant as the (9*Z*) isomer.

The comparison between the relative proportions of lycopene isomers, between smokers and non-smokers, revealed that there was a significant difference (*p* < 0.05, ANOVA) in the ratio of total-*Z*:all-*E* lycopene, between non-smokers (1.52 ± 0.38:1.00) and smokers (1.85 ± 0.29:1.00). For the individual geometric isomers, a significant difference (*p* < 0.05, ANOVA) was only seen for the fractions of the (13*Z*)- and (all-*E*)-lycopene isomers in the plasma of smokers and non-smokers, i.e., there was a rise in the relative abundance of (13*Z*)-lycopene and an accompanying decrease in the level of (all-*E*)-lycopene, in the smoking cohort. There was no significant difference in the relative proportions of the two other lycopene isomers found in human plasma from smokers and non-smokers.

## 4. Discussion

Gaseous phase cigarette smoke is known to cross the alveolae in the lungs [10,12], where it can interact with circulating plasma antioxidants, resulting in their depletion [17]. In this study, an increased circulation concentration of oxidised LDL (81.9 ± 9.05 vs. 55.7 ± 12.8 IU/L) was observed in smokers. The presence of oxidised LDL is thought to be a combination of oxidised lipids, which through peroxidation reactions can lead to oxidation of the apo B-100 protein. Alternatively, certain radicals can rapidy oxidise apo100 in LDL particles directly [21]. The smokers’ data also indicated atherogenic profiles (Table 1), with increased total cholesterol (6.19 ± 0.38 vs. 5.2 ± 0.68 mmol/L) and lower levels of HDL (1.31 ± 0.17 vs. 1.59 ± 0.5 mmol/L). 

A meta-analysis by Alberg [17], examining studies measuring antioxidant levels in smokers and non-smokers, found that ascorbic acid and the provitamin A carotenoids (β-carotene, α-carotene and β-cryptoxanthin) were depleted by ca. 25% in smokers. They suggested that cigarette smoke itself depletes certain antioxidants, including ascrobic acid and pro-vitamin A carotenoids, but also recognised that most smokers had a reduced intake of these antixodants in their diet. However, the association between levels of α-tocopherol and the non-provitamin A carotenoids (lycopene, zeaxanthin and lutein) and smoking was much weaker (5% in the case of lycopene). In the current study, plasma concentrations of both β-carotene and lycopene were not significantly different between smokers and non-smokers. It is not known whether there were differences in dietary habits between smokers and non-smokers in this study, which had a relatively small sample size compared to many earlier studies involving dietary changes, cited by Alberg [17]. Instead, we observed differences in the relative proportions of some geometrical isomers of lycopene between those participants who smoked and those who did not. In tomatoes and tomato-based products, (all-*E*)-lycopene may account for up to 97% of total lycopene [22] but the levels of isomers may be modified by food processing, especially cooking [23]. In plasma, the relative abundance of (*Z*)-isomers of lycopene increases compared to the (all-*E*) form. During the digestion process, it is thought that the incorporation of lycopene (*Z*)-isomers into micelles is preferred to the (all-*E*) isomer [23]. This view has recently been challenged by Moran et al. [24] who used ^13^C-lycopene and found that the incorporation of (*Z*)-isomers into micelles was not a factor, and that the enterocyte had no role in influencing the relative abundancies of different geometric isomers of lycopene in circulation. Fast-turnover tissues, such as the liver, that could influence the proportion of (*Z*)-isomers in circulation were identified [24]. 

Additional factors, especially free radical attack, may influence the isomerisation of carotenoids [25] When isolated human plasma and isolated LDL particles containing dietary carotenoids, such as β-carotene and lycopene, were exposed to cigarette smoke (or Sin-1), the amounts of these carotenoids declined. Free radical attack is also known to bring about geometrical isomeration of lycopene [18,19,20]. Alongside the oxidative degradation of these carotenoids, due to exposure to cigarette smoke, there were also substantial changes to the relative amounts of some geometrical isomers. In general, (all-*E*) to (*Z*) isomerisation was promoted under such conditions. Particularly noticeable, however, was the formation of (13*Z*)-lycopene as the major conversion project that accompanied the depletion of (all-*E*) lycopene [19]. This was generated in preference to the (5*Z*)-isomer, which has been determined to be more thermodynamically stable e.g., [26]. 

In the current study, the ratio of (13*Z*):total lycopene was significantly different in smokers (0.84:1.00) compared to non-smokers (1.04:1.00; see Figure 3). Richelle et al. [23] demonstrated that the preparation of tomato products prior to ingestion has no impact on the isomerisation of lycopene occurring during absorption in humans—the (9*Z*) and (13*Z*) isomers were less well absorbed or converted into the more stable (all-*E*) and (5*Z*) forms. It is therefore unlikely that the differences seen here between the isomeric composition of lycopene in smokers and non-smokers are due to differences in their dietary intake of tomato products. Instead, it is proposed that gaseous phase cigarette smoke could induce isomerisation of (all-*E*)-lycopene in vivo, as the result of increased peroxidation within the LDL particles. The plasma profiles of smokers did not reveal the presence of any oxidative intermediates of lycopene (e.g., lycopene-5,6-epoxide and 2,6-cyclolycopene-1,5-diol) that have been seen in in vitro studies as the result of exposure to cigarette smoke [19]. Such intermediates have only been seen to date in circumstances where reactions between lycopene and smoke have been considerably slowed (by cooling), and even then are present at very low levels, making detection challenging (e.g., requiring the use of electrochemical detection or MS–MS, techniques not used in the present study). As such, these would not necessarily be expected to be detected in vivo. The different geometric isomers will themselves degrade under such oxidative conditions, and no specific data is available regarding the relative rates for this. However, the fact that the relative propotion of the least stable [26] of the isomers detected in this study (i.e., the 13*Z* form) increased, suggests that this is not a major factor.

## 5. Conclusions

This study observed that the ratio of total-*Z*: all-*E* lycopene in smokers is significantly higher to that seen in non-smokers. Analysis of the individual geometric isomers of this carotenoid showed elevated levels of *Z*-isomers relative to the (all-*E*) form. A significant difference in the relative levels of (13*Z*)-lycopene was seen when comparing smokers with non-smokers. This supports the view that, in smokers, the (13*Z*)-isomer is generated from the (all-*E*) form in preference to the formation of the more thermodynamically stable (5*Z*) and (9*Z*)-isomers. This is in agreement with observations previously made in vitro regarding the preferred generation of the 13*Z*-isomer when lycopene is exposed to cigarette smoke [18,19]. The results suggest that the relative amounts of (13*Z*)-lycopene could be used as an indicator of oxidative damage to lycopene in vivo. However, further, larger-scale, in vivo studies are needed in order to confirm these results and better understand the interactions of lycopene with cigarette smoke and its components.

## Figures and Tables

**Table 1:** Clinical and biochemical profiles of the smoking and non-smoking groups (*n* = 52 ± SD). * indicates significant differences (*p* < 0.05) between the smoking and non-smoking cohorts.

Parameter	Smoking	Non-Smoking	*p*-Value	Normal Range
Age (y)	43.3 ± 8.9	42.3 ± 14.3		
BMI (kg/m^2^)	27.11 ± 4.15	25.9 ± 3.54		<25
Male	12	15		
Female	13	12		
Cigarettes smoked per day	14.6 ± 2.4	0		
Total cholesterol (mmol/L)	6.19 ± 0.38 *	5.2 ± 0.68	0.0006	<5.17
LDL-cholesterol (mmol/L)	3.38 ± 0.34 *	2.81 ± 0.55	0.0004	<2.6
HDL-cholesterol (mmol/L)	1.31 ± 0.17	1.59 ± 0.5	0.1690	>1.04
Ratio of total cholesterol:HDL cholesterol	4.73 ± 0.64 *	3.27 ± 1.10	0.0140	<4.5
Plasma oxLDL (IU/L)	81.9 ± 9.05 *	55.7 ± 12.8	0.0070	
Triglycerides (mmol/L)	2.52 ± 0.76 *	1.61 ± 0.87	0.0150	<1.69

**Table 2:** Levels of β-carotene and lycopene in plasma isolated from smoking and non-smoking participants (*n* = 52 ± SD) determined by HPLC. * indicates significant differences between smokers and non-smokers (*p* < 0.05).

Carotenoid (μmol/L)	Smoking	Non-Smoking	*p*-Value
β-Carotene	0.47 ± 0.22	0.54 ± 0.40	0.470
Total lycopene	0.71 ± 0.30	0.58 ± 0.28	0.106
(all-*E)*-lycopene	0.25 ± 0.02 *	0.29 ± 0.04 *	0.018
(5*Z*)-lycopene	0.30 ± 0.02	0.27 ± 0.04	0.070
(9*Z*)-lycopene	0.06 ± 0.01	0.05 ± 0.01	0.180
(13*Z*)-lycopene	0.11 ± 0.01 *	0.10 ± 0.04 *	0.030

**Table 3:** On-line spectral characteristics and retention times, determined by diode-array detection (in the range of 200–600 nm) using HPLC separation with a C30 reversed-phase column (see Graham et al. [18] for further details). λ_max_—wavelength of the main absorption bands; A_B_/A_II_—the relative intensities of the heights of the *cis*-peak and the middle absorption band (λ_max_); T_R_—the retention time.

Geometric Isomer	λ_max_ (nm)				A_B_/A_II_	T_R_ (min)
(all-*E*)-lycopene		446	472	503	--	29.5
(5*Z*)-lycopene		446	472	503	--	32.0
(9*Z*)-lycopene	362	440	466	498	0.68	20.7
(13*Z*)-lycopene	360	442	466	498	0.58	16.3
